# Effects of RAP Asphalt Mortar Migration on the Cracking Performance of Recycled Asphalt Mixtures: Evaluation, Mechanism, and Regulation Strategy

**DOI:** 10.3390/ma19204399

**Published:** 2026-10-19

**Authors:** Yuquan Yao, Jiayu Liu, Jiayun Xu, Wei Zhang, Jie Gao, Kai Liu, Jiangang Yang

**Affiliations:** 1 State Key Laboratory of Safety and Resilience of Civil Engineering in Mountain Area, East China Jiaotong University, Nanchang 330013, China; 3688@ecjtu.edu.cn (Y.Y.); 3738@ecjtu.edu.cn (J.X.); gaojie@ecjtu.edu.cn (J.G.); 2 Jiangxi Provincial Key Laboratory of Traffic Infrastructure Safety, East China Jiaotong University, Nanchang 330013, China; 3 School of Civil Engineering and Architecture, East China Jiaotong University, Nanchang 330013, China; 2025018085901033@ecjtu.edu.cn (J.L.); 2025019081400001@ecjtu.edu.cn (W.Z.); 4 School of Transportation and Civil Engineering, Nantong University, Nantong 226019, China; ronaldo_1@163.com; 5 School of Automotive and Traffic Engineering, Hefei University of Technology, Hefei 230009, China

**Keywords:** plant-mixed hot recycled asphalt mixtures (PHRAMs), reclaimed asphalt pavement (RAP), RAP particle dispersion, cracking performance

## Abstract

The dispersion behavior of reclaimed asphalt pavement (RAP) particles during hot mixing directly affects the blending of aged and virgin materials and consequently influences the cracking performance of plant-mixed hot recycled asphalt mixtures (PHRAMs). This study investigated the effects of material composition and mixing process parameters on RAP particle dispersion and cracking performance of PHRAMs. PHRAMs containing 40% RAP were prepared using an orthogonal experimental design considering gradation type, asphalt binder content, mixing time, and RAP preheating temperature. Using a developed magnetite tracer method, the RAP asphalt mortar migration rate index was calculated from the mass of adhering mineral particles smaller than 2.36 mm, normalized by tracer aggregate mass and expressed relative to the corresponding loading in a disintegrated-RAP reference mixture. Semi-circular bending tests were conducted to evaluate intermediate-temperature crack propagation and low-temperature cracking resistance. Finally, the relationship between the RAP asphalt mortar migration rate and cracking performance was comprehensively analyzed. The results showed that gradation type and asphalt binder content were the dominant factors affecting RAP asphalt mortar migration, whereas asphalt binder content was the key factor governing cracking performance. Although RAP asphalt mortar migration was correlated with cracking resistance, it was not the primary controlling factor. Comprehensive analysis indicated that asphalt film thickness and gradation characteristics were more closely associated with cracking performance, together with the effects of RAP asphalt mortar migration and aged asphalt binder proportion. Accordingly, a multi-objective optimization framework was proposed to regulate RAP particle dispersion under cracking performance constraints and determine reasonable ranges of material composition and mixing process parameters.

## 1. Introduction

Reclaimed asphalt pavement (RAP) has been increasingly used in asphalt pavement construction because of its advantages in conserving natural resources, reducing energy consumption, and lowering construction costs [1,2,3]. In particular, plant-mixed hot recycled asphalt mixtures (PHRAMs) provide an effective approach for incorporating a relatively high proportion of RAP into asphalt mixtures [4]. However, previous studies [5,6,7] have shown that RAP particles exhibit pronounced agglomeration characteristics, and the aged asphalt mortar coating their surfaces mainly undergoes four states during hot mixing: flowing aged asphalt, softened aged asphalt, hardened aged asphalt, and absorbed aged asphalt. Therefore, the incorporation of RAP introduces aged asphalt binder and agglomerated RAP particles into the mixture, making the material system more heterogeneous than conventional asphalt mixtures [8,9]. The interaction among aged asphalt binder, virgin asphalt binder, rejuvenator, and aggregates during hot mixing has an important influence on the internal structure and engineering performance of PHRAMs [10].

RAP particle dispersion during hot mixing involves changes in agglomeration and the redistribution of aged mortar within the PHRAM. Ai et al. [6] reviewed RAP agglomeration and partial blending, emphasizing that both aggregate gradation and effective binder content require consideration in recycled mixture design. He et al. [10] quantified inherent and relative agglomeration rates using gradation curves and examined their evolution under different mixing conditions, together with their implications for low-temperature cracking and other mixture properties. Their results also showed that fine RAP particles could re-agglomerate during mixing. These findings demonstrate that RAP dispersion is a dynamic process. However, gradation-based agglomeration indicators do not directly quantify mortar transfer or aged–virgin binder diffusion, and their relationship with fracture response requires interpretation alongside changes in mixture composition.

Aged binder mobilization and availability provide another perspective on the participation of RAP in the recycled material system. Lo Presti et al. [5] distinguished binder activity, availability, and degree of blending within a common conceptual framework. Kaseer et al. [11] proposed a binder availability factor to support adjustments to virgin binder content, while Ma et al. [12] reviewed the mechanisms, measurement approaches, and applications of reclaimed binder availability and blending efficiency. At the mortar scale, Gao et al. [13] quantified RAP mastic migration during hot mixing and investigated the effects of material and processing parameters. More recently, Zhang et al. [14] combined chemical and microscopic characterization to examine coupled mastic mobilization and blending, showing that extensive mobilization did not necessarily result in complete blending. Collectively, these studies indicate that binder availability, mortar migration, and binder blending describe related but distinct quantities. In particular, mortar transfer to virgin aggregate surfaces provides an indirect measure of RAP dispersion rather than a direct measure of molecular blending or mixture-wide spatial uniformity.

Research on blending mechanisms has employed complementary chemical, rheological, microscopic, and numerical approaches [15,16,17,18,19,20,21,22,23]. Bowers et al. [15] investigated RAP blending using rheological properties and molecular weight distributions, while Yao et al. [16] used Fourier transform infrared spectroscopy to evaluate blending under different material compositions and mixing conditions. Zhang et al. [17] reviewed diffusion and blending theories, models, and characterization methods, highlighting differences in their measurement principles and applicability. Molecular dynamics simulations have further related interfacial diffusion and blending states to binder fracture behavior [18]. These approaches provide valuable information on binder interaction, but the measured quantities depend on the observation scale and experimental procedure. Chemical composition and binder-level mechanical responses alone cannot fully describe the redistribution of mortar, aggregate arrangement, or fracture behavior of the recycled mixture. Accordingly, binder-scale evidence needs to be complemented by mixture-scale measurements.

Previous studies have also established links between RAP agglomeration, blending, and cracking resistance [18,19,20,21,22,23,24,25,26,27,28,29]. Cui et al. [18] found that cracks in partially blended binder models initiated within the blending zone and that greater blending was associated with higher binder fracture energy. At the mixture scale, He et al. [10] evaluated the influence of RAP agglomeration on low-temperature cracking, while Tao et al. [19] investigated blending and diffusion together with low-temperature fracture response under different processing and conditioning conditions. The latter study showed that the relationship depended on the parameter being varied: conditions promoting blending could also promote additional aging and thereby impair cracking resistance. These findings support the relevance of blending and dispersion to fracture behavior, but also caution against assuming a universally monotonic relationship. Furthermore, binder content and gradation changes alter mixture characteristics that may influence cracking independently of the measured extent of mortar transfer.

Building on these advances, a specific issue requiring further examination is whether RAP asphalt mortar migration can serve as a reliable stand-alone indicator of cracking performance when material composition and mixing conditions vary. Binder content and gradation affect asphalt film thickness and the relative proportions of aged and virgin binder, whereas mixing conditions influence mortar redistribution and thermal history. Consequently, an observed migration–fracture relationship may reflect several concurrent changes. The research gap addressed here concerns the correspondence between mortar migration and mixture fracture response, and the limitations of using migration rate alone to interpret cracking performance across different combinations of design and processing parameters.

To address this question, the present study combines the previously established RAP asphalt mortar migration method [13] with semi-circular bending tests in a common orthogonal experimental framework. Gradation type, asphalt binder content, mixing time, and RAP preheating temperature are varied while RAP content and rejuvenator dosage are held constant. The analysis compares the factors governing mortar migration with those governing intermediate-temperature fracture behavior and low-temperature cracking resistance, and examines migration alongside asphalt film thickness, aged binder proportion, and gradation index. The contribution is to clarify the explanatory value and limitations of mortar migration within the investigated experimental domain. A conceptual framework is subsequently proposed for regulating RAP dispersion subject to cracking-performance requirements.

## 2. Materials and Methodology

### 2.1. Raw Materials

The raw materials used in this study included RAP, virgin aggregates, styrene–butadiene–styrene (SBS) modified asphalt binder, and a rejuvenating agent.

**Figure 1:**
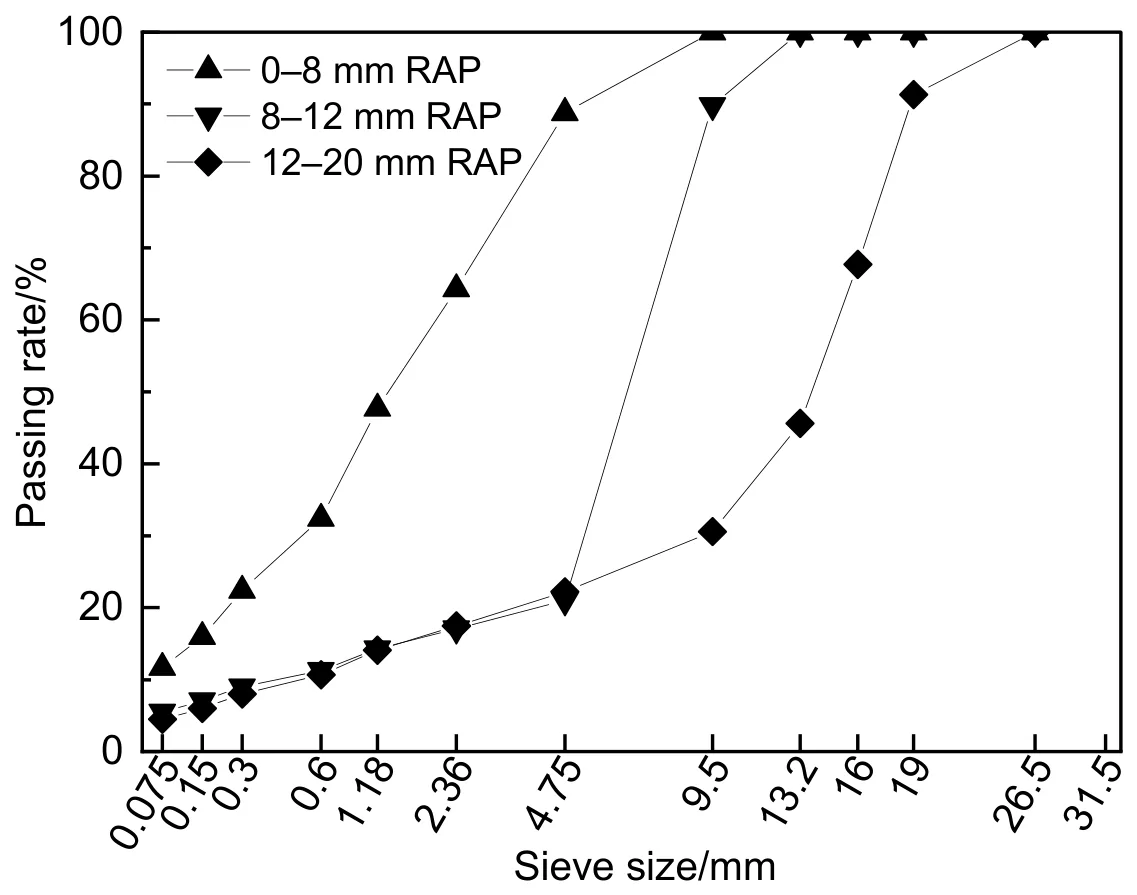
Test results of aggregate gradation in RAP.

The RAP was obtained by milling from the Wenjiazhen–Shatang’ai section of the Fuzhou–Yinchuan Expressway in Jiangxi Province, China. After crushing and screening, the RAP was classified into three particle-size fractions, namely 0–8 mm, 8–12 mm, and 12–20 mm, for subsequent recycling. The aggregate gradation and asphalt binder content of the RAP were determined using extraction and sieve analysis. The results are presented in Figure 1. The asphalt binder contents of the three RAP fractions were 5.64%, 2.87%, and 2.70%, respectively. The aged asphalt binder was separated from the solvent in the asphalt solution extracted from the RAP using a rotary evaporator. Subsequently, the basic properties of the recovered aged asphalt binder and the properties of the aggregates contained in the RAP were evaluated. The corresponding test results are summarized in Table 1.

The virgin aggregates used in this study were limestone, with nominal size ranges of 0–5 mm, 5–10 mm, and 10–20 mm. The basic properties of the aggregates and the mineral filler are presented in Table 2. To facilitate the mix design of the PHRAM, the three aggregate fractions were further separated into individual size fractions according to sieve size using the washing and sieving method.

The RA-102 rejuvenating agent used in this study was a pale-yellow liquid with a density of 0.835 g/cm^3^ at 15 °C and a viscosity of 0.76 Pa·s at 20 °C. Component analysis showed that the rejuvenating agent contained 21.3% saturates and 63.4% aromatics, with aromatics being the predominant component.

### 2.2. Experimental Design

**Figure 2:**
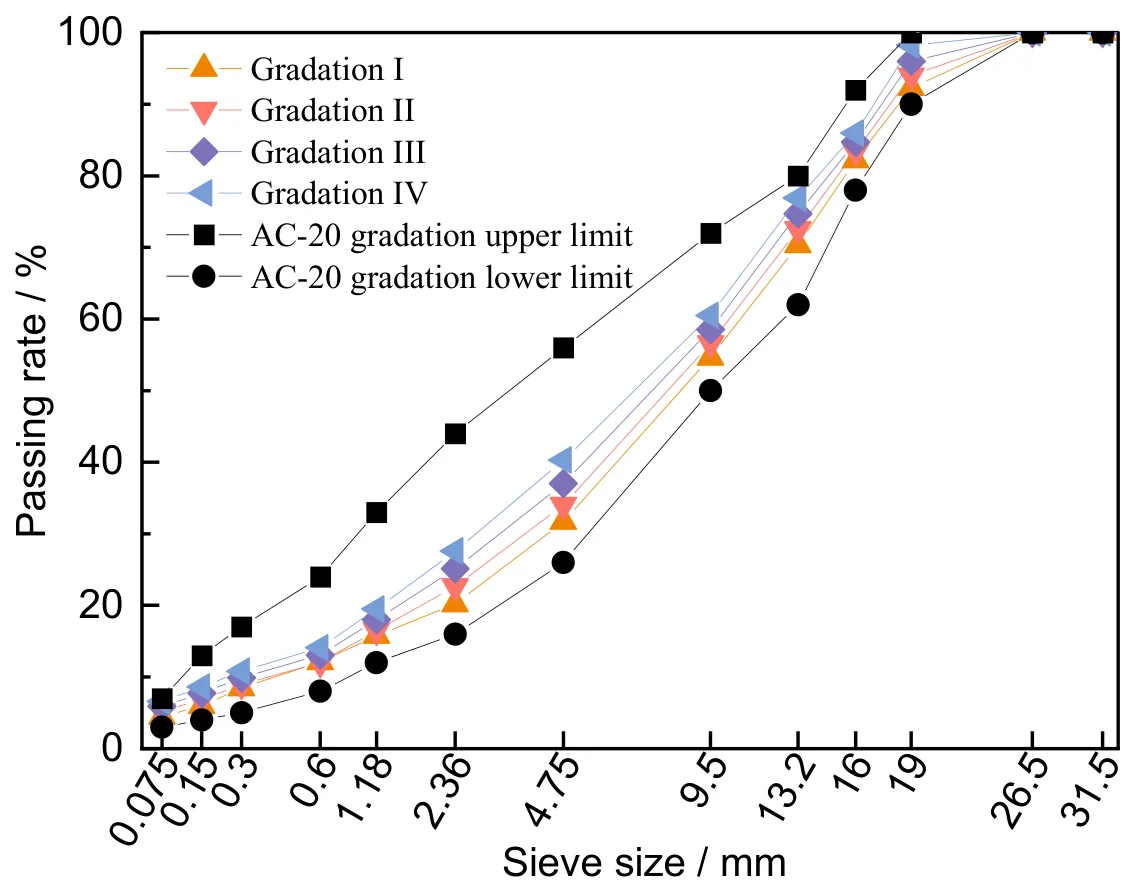
Four designed gradation types for AC-20.

The design of PHRAMs requires consideration of both material composition and mixing process parameters. The material-related parameters commonly considered include gradation type, rejuvenator dosage, asphalt binder content, and RAP content, whereas the mixing process parameters mainly include RAP preheating temperature, mixing time, mixing temperature, and mixing sequence. According to field investigations, the RAP content, rejuvenator dosage, mixing temperature, and other mixing process parameters are generally maintained at relatively fixed levels during the actual production of PHRAMs. Therefore, this study focused on the effects of gradation type, asphalt binder content, RAP preheating temperature, and mixing time. PHRAM was designed as an AC-20 asphalt mixture. For the gradation type factor, four levels were considered, corresponding to Gradations I, II, III, and IV, respectively. The four gradation types adopted in the mix design are shown in Figure 2. The asphalt binder content was varied at four levels of 3.7%, 4.0%, 4.3%, and 4.6%, while the mixing time was set to 60, 120, 150, and 180 s. Three levels of RAP preheating temperature, i.e., 90, 110, and 130 °C, were considered. These factor–level combinations were designed to systematically investigate the effects of material composition and mixing process parameters on the performance of recycled asphalt mixtures.

Based on these factors and the levels, an orthogonal experimental design was employed to investigate their effects on the migration behavior of RAP asphalt mortar and the cracking resistance of PHRAMs. The detailed experimental scheme is presented in Table 3.

During the preparation of the PHRAM, the RAP content was fixed at 40% by mass of the total aggregate. Maintaining a fixed RAP dosage provides a defined background for comparing the investigated material and processing parameters without introducing RAP content as an additional experimental factor. The PHRAM mixing temperature was maintained at 160 °C. When the rejuvenator dosage was 5.0% by mass of the aged asphalt binder, the penetration of the aged binder at 25 °C could be restored to a level comparable to that of the virgin binder. Therefore, the rejuvenator dosage was set at 5.0% by mass of the aged asphalt binder. The mixing sequence was as follows: RAP was first premixed with the rejuvenator, followed by the addition and mixing of virgin aggregates and virgin asphalt binder, and finally the mineral filler was added and mixed. Under the above experimental conditions, PHRAMs were prepared according to the experimental scheme shown in Table 3 for subsequent investigation.

### 2.3. Experimental Methods

#### 2.3.1. Evaluation Method for RAP Asphalt Mortar Migration Rate

Based on our previous study [13], the degree of aged asphalt migration in PHRAMs can be evaluated using the RAP asphalt mortar migration rate. The method uses RAP-derived fine mineral particles adhering to magnetite tracer aggregates as an indirect measure of mortar transfer. The resulting RAP asphalt mortar migration rate is an operational index obtained by comparing the tracer-surface fine-particle loading in the experimental mixture with that in a disintegrated-RAP reference mixture. The proposed evaluation method mainly consists of three steps. In addition, the RAP asphalt mortar migration rate test was conducted in duplicate, and the average of the results was taken as the representative value.

Step 1: Determination of the mass of asphalt mortar coating the tracer aggregate in the experimental group.

According to the designed experimental scheme, PHRAMs were prepared using 0–20 mm RAP. To accurately distinguish the source of the asphalt mortar adhering to the surface of the virgin aggregates, virgin aggregates with particle sizes ranging from 2.36 to 26.5 mm were used. Among them, the 16–26.5 mm fraction was replaced with magnetite, which served as the tracer aggregate. After mixing, the magnetite particles were separated using a 16 mm sieve to eliminate interference from RAP aggregates within the same particle-size range. Subsequently, the mass of particles smaller than 2.36 mm adhering to the surface of the magnetite was determined using the ignition method followed by wet sieving. This value was used to characterize the extent of migration of aged asphalt mortar from the RAP surface to the surface of the virgin aggregates during hot mixing. In the test, the mass of magnetite was denoted as *m*_1_, and the mass of RAP aggregate particles smaller than 2.36 mm adhering to its surface was denoted as *k*_1_. The concentration of aged asphalt mortar (*λ*_1_) in the experimental group was then calculated according to Equation (1).

(1) $$\lambda_{1}=\frac{k_{1}}{m_{1}}\times 100\%$$

Step 2: Determination of the mass of asphalt mortar coating the tracer aggregate in the control group.

The control group was used to determine the level of asphalt mortar coating on the aggregate surface under ideal conditions. The same RAP as that used in the experimental group was employed, and the aged asphalt binder and aggregates in the RAP were separated in advance to simulate the complete disintegration of RAP. The separated RAP aggregates were premixed with the virgin aggregates, after which the virgin asphalt binder was added for mixing. The virgin aggregates and virgin asphalt binder used in the control group were identical to those used in the experimental group. After mixing, the magnetite particles were separated using the same procedure as that adopted for the experimental group. The mass of the magnetite was denoted as *m*_2_, and the mass of particles smaller than 2.36 mm adhering to its surface was denoted as *k*_2_. Finally, the asphalt mortar concentration (*λ*_2_) on the aggregate surface under the ideal condition was calculated according to Equation (2).

(2) $$\lambda_{2}=\frac{k_{2}}{m_{2}}\times 100\%$$

Step 3: Calculation of the RAP asphalt mortar migration rate.

The effect of RAP agglomeration on the migration of aged asphalt mortar was evaluated by comparing the asphalt mortar concentrations on the surfaces of the virgin aggregates in the experimental and control groups. The migration rate (*λ*) of the aged asphalt mortar was then calculated according to Equation (3).

(3) $$\lambda =\frac{\lambda_{1}}{\lambda_{2}}\times 100\%$$

Excluding virgin aggregates smaller than 2.36 mm reduces interference from virgin fine aggregates in the tracer measurement, but introduces a difference between the test configuration and the complete mixture. Virgin fines provide additional surfaces for binder and mortar coating and may alter mortar distribution, interparticle contact, and mixing behavior. Their exclusion can therefore change the amount of tracked material deposited on the magnetite surfaces. The measurement obtained under this restricted aggregate configuration should not be assumed to equal that obtained in a complete mixture. Normalization against the disintegrated-RAP reference does not necessarily remove this effect. Accordingly, the reported values are interpreted as normalized fine-particle transfer indices used as proxies for RAP mortar migration under the specified test conditions.

Overall, this method merely provides a relatively convenient technical approach to indirectly characterize the migration of aged asphalt on RAP particle surfaces during hot mixing. There may be some deviation from actual results, but this is considered a systematic error and does not affect our investigation into how different factors influence migration patterns.

#### 2.3.2. Evaluation of Mixture Cracking Performance Based on the SCB Test

PHRAMs were prepared according to the designed experimental scheme, and their cracking performance was evaluated using the semi-circular bending (SCB) test. For specimen preparation, two cylindrical specimens with a diameter of 150 mm and a height of 150 mm were compacted using a gyratory compactor. After cooling for 24 h, two 50 mm thick cylindrical slices were cut from the central portion of each compacted specimen. Each slice was then cut along its diameter into two semicircular specimens, resulting in a total of eight semicircular specimens. Subsequently, a prefabricated notch with a depth of 15 mm and a width of 1.5 mm was cut at the midpoint of the flat edge of each semicircular specimen. The prepared SCB specimens were used to evaluate low-temperature cracking resistance and crack propagation resistance, respectively. For each experimental scheme, eight specimens were prepared for each of the two test conditions.

(1) Crack propagation performance test

The semi-circular bending (SCB) test was conducted using an SYD-0730A multifunctional automatic asphalt pressure testing machine to evaluate the resistance of asphalt mixtures to further crack propagation under traffic loading after crack initiation. The test temperature was maintained at 25 °C, and the loading rate was set at 50 mm/min. Before testing, an initial load of 0.1 kN was applied to each specimen. The specimen was then subjected to linear loading at the prescribed rate, and the test was terminated when the load decreased below 0.1 kN.

A typical load–displacement curve can be divided into two stages: crack initiation and crack propagation. The areas under the curve before and after the peak load, denoted as Area I and Area II, respectively, are used to characterize the fracture energy associated with the corresponding stages. The sum of the two fracture energy components represents the overall cracking resistance of the mixture, with a higher fracture energy indicating better cracking resistance. In addition, the Flexibility Index (FI) was adopted to evaluate the crack propagation performance of the mixture. A higher FI value indicates a greater ability of the mixture to resist crack propagation and accommodate deformation. The fracture energies before and after the peak load, as well as the FI, were calculated according to Equations (4)–(6).

(4) $$G_{f}^{I}=\frac{W_{f}^{I}}{(r-a)\times t}\times 10^{6}$$

(5) $$G_{f}^{\mathrm{II}}=\frac{W_{f}^{\mathrm{II}}}{(r-a)\times t}\times 10^{6}$$

(6) $$\mathrm{FI}=\frac{G_{f}^{I}+G_{f}^{\mathrm{II}}}{\left|m\right|}\times A$$

where $G_{f}^{I}$ and $G_{f}^{\mathrm{II}}$ represent the fracture energies corresponding to Area I before the peak load and Area II after the peak load, respectively, J/m^2^; $W_{f}^{I}$ and $W_{f}^{\mathrm{II}}$ represent the fracture work corresponding to Area I before the peak load and Area II after the peak load, respectively, J; FI represents the Flexibility Index; r, a, and t represent the radius of the semicircular specimen, notch length, and specimen thickness, respectively, mm; m represents the post-peak slope determined at the inflection point of the load–displacement curve after the peak load, kN/mm; A represents the unit conversion and scaling factor, with a value of 0.01.

(2) Low-temperature cracking resistance test

The low-temperature cracking resistance of the PHRAMs was evaluated using the SCB test. The test temperature was set at −10 °C. Prior to testing, the specimens were conditioned at this temperature for 4–6 h to ensure that their internal temperature reached the specified test temperature. Based on the findings reported by Feng et al. [31], the specimens were loaded under displacement control by applying a constant downward displacement at the top of the specimen. The loading rate was set at 1.2 mm/min, and loading was continued until specimen failure occurred. Under low-temperature conditions, the semicircular specimens exhibited predominantly brittle fracture behavior.

The low-temperature cracking resistance of the PHRAMs was evaluated in terms of peak load, fracture energy, fracture toughness, and stiffness. The fracture energy was determined from the area under the load–displacement curve before the peak load and calculated according to Equation (7). The fracture toughness was determined based on the *K*-criterion for Mode I fracture and calculated according to Equations (8)–(10). The stiffness was defined as the slope of the linear ascending portion of the load–displacement curve.

(7) $$G_{f}=\frac{W_{f}}{(r-a)\times t}\times 10^{6}$$

(8) $$K_{IC}=Y_{I(0.8)}\times \sigma_{0}\times \sqrt{\mathrm{\pi a}}$$

(9) $$\sigma_{0}=\frac{P_{c}}{2\mathrm{rt}}$$

(10) $$Y_{I(0.8)}=4.782+1.219\left(\frac{a}{r}\right)+0.063e^{7.045\left(\frac{a}{r}\right)}$$

where *G_f_* represents the fracture energy, J/m^2^; *W_f_* represents the fracture work, J; *K*_IC_ represents the fracture toughness, MPa/m; *Y* represents the normalized stress intensity factor; *σ*_0_ represents the stress corresponding to the peak load, MPa; *P*_c_ represents the peak load, kN.

By recording the load–displacement data during the loading process, the low-temperature cracking resistance of the PHRAMs was evaluated in terms of fracture energy, fracture toughness, and stiffness. Higher fracture energy and fracture toughness indicate a greater capacity of the material to absorb energy and resist crack propagation, whereas the stiffness index is used to characterize the resistance of the material to deformation.

## 3. Results and Discussion

### 3.1. Evaluation of RAP Asphalt Mortar Migration Rate

**Figure 3:**
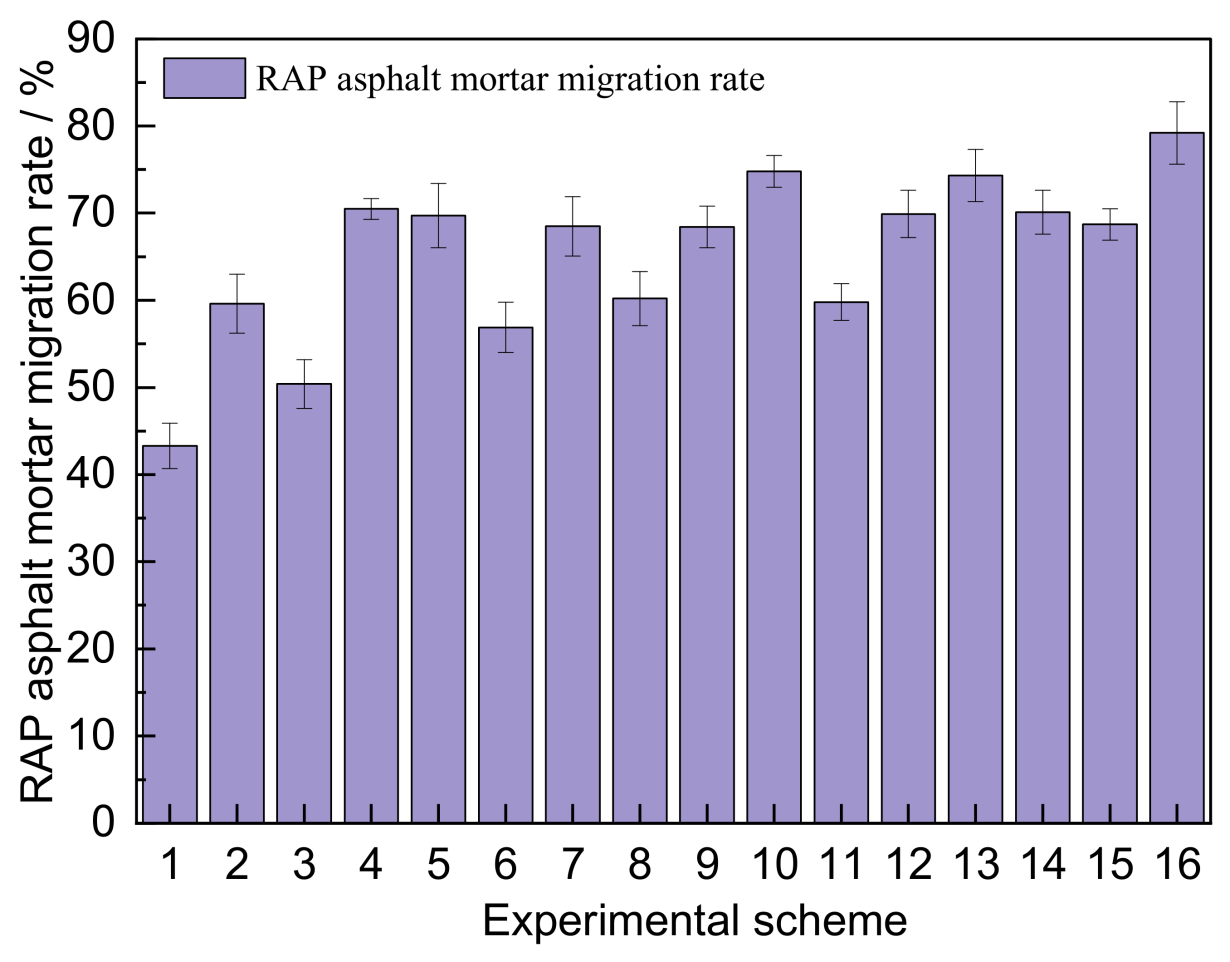
Test results of RAP asphalt mortar migration rate.

The RAP asphalt mortar migration rates were calculated for each experimental scheme, and the results are presented in Figure 3. Based on the results shown in Figure 3, range analysis and analysis of variance (ANOVA) were further conducted to evaluate the effects of the experimental factors on the RAP asphalt mortar migration rate. The corresponding results are presented in Table 4 and Table 5, respectively.

As shown in Table 4, the RAP asphalt mortar migration rate exhibited an increasing trend as the gradation of the PHRAM changed from coarse to fine, the asphalt binder content increased, the mixing time was prolonged, and the RAP preheating temperature increased. A finer gradation increases the specific surface area of the aggregates and enhances interparticle contact. A higher asphalt binder content and a longer mixing time promote more sufficient contact between the RAP asphalt mortar and the virgin aggregates and virgin asphalt binder. In addition, increasing the RAP preheating temperature reduces the viscosity of the aged asphalt binder and weakens its adhesion to the aggregate surface, thereby facilitating the migration of RAP asphalt mortar.

As shown in Table 5, the ANOVA model yielded an (*R*^2^) value of 0.986, indicating a high goodness of fit and confirming the validity of the ANOVA results. Based on the calculated F-values, gradation type and asphalt binder content had highly significant effects on the RAP asphalt mortar migration rate, whereas mixing time and RAP preheating temperature had significant effects. These results demonstrate that all four factors significantly influenced the RAP asphalt mortar migration rate.

In summary, both the range analysis and ANOVA yielded the same ranking of the effects of the four factors: gradation type > asphalt binder content > mixing time > RAP preheating temperature. These results indicate that material composition factors exert a more pronounced influence on the RAP asphalt mortar migration rate in PHRAMs than mixing process factors.

### 3.2. Evaluation of Crack Propagation Performance

During the loading of the SCB specimen, the specimen initially underwent a stable loading stage. When the load reached approximately 80% of the ultimate load, microcracks began to initiate at the central notch. Subsequently, the crack continuously propagated along the notch and gradually extended through the specimen until complete failure occurred. Loading was terminated when the load decreased below 0.1 kN.

Based on the load–displacement data recorded throughout the loading process, parameters including the peak load (*F*_max_), pre-peak ($G_{f}^{I}$) and post-peak ($G_{f}^{\mathrm{II}}$) fracture energies, total fracture energy (*Gf*), post-peak slope (*m*), and Flexibility Index (*FI*) were calculated to evaluate the effects of material composition factors on the crack propagation performance of the PHRAMs. The calculated results are presented in Table 6.

The results presented in Table 6 indicate that gradation and asphalt binder content exert pronounced effects on the crack propagation performance of PHRAMs. As the gradation became finer, both the peak load and the cracking resistance during the crack initiation stage increased. In contrast, mixtures with coarser gradations generally exhibited higher post-peak fracture energy, total fracture energy, and Flexibility Index, indicating greater resistance to crack propagation. With increasing asphalt binder content, the cracking resistance of the mixtures improved during both the crack initiation and crack propagation stages. Overall, a coarser gradation combined with a higher asphalt binder content is beneficial for enhancing the crack propagation resistance of PHRAMs.

To avoid inconsistencies among the range analysis results obtained using multiple cracking performance indicators, Pearson correlation analysis was conducted to examine the correlations between *Gf* and the other performance indicators, with correlation coefficients of −0.2539, 0.7043, 0.9504, 0.5592, and 0.7610 for the *F*_max_, $G_{f}^{I}$, $G_{f}^{\mathrm{II}}$, *m*, and *FI*, respectively. Since *Gf* shows good correlation with other indicators and encompasses the entire process of crack initiation and propagation, it is selected as a comprehensive indicator for evaluating the crack propagation resistance of PHRAMs. Based on the *Gf* test results, range analysis and ANOVA were performed, and the corresponding results are presented in Table 7 and Table 8, respectively.

As shown in Table 7, the total fracture energy (*Gf*) of the PHRAMs initially increased and then decreased as the gradation changed from coarse to fine and as the mixing time increased. In contrast, the *Gf* generally exhibited an increasing trend with increasing asphalt binder content and RAP preheating temperature. As the mixing time increased, the *Gf* initially improved and then decreased, which may be related to excessive migration of RAP asphalt mortar. With increasing amounts of high-modulus aged asphalt mortar migrating and becoming incorporated into the mixture system, the overall asphalt mortar gradually became stiffer [32,33]. Although a rejuvenator was added, its restorative effect may have been limited, thereby leading to a reduction in the crack propagation resistance of the PHRAMs. The range analysis results indicate that the effects of the investigated factors on *Gf* followed the order: asphalt content > RAP preheating temperature > gradation type > mixing time, with asphalt binder content exerting the most pronounced influence. With the objective of achieving a higher fracture energy, the optimal levels of the four factors were determined to be Gradation II, an asphalt binder content of 4.6%, a mixing time of 150 s, and an RAP preheating temperature of 130 °C.

As shown in Table 8, the ANOVA results indicate that asphalt binder content and RAP preheating temperature had highly significant effects on *Gf*, whereas gradation type and mixing time had significant effects. Combined with the range analysis results, the influence of the four factors on *Gf* followed the order: asphalt content > RAP preheating temperature > gradation type > mixing time. Among these factors, asphalt binder content was identified as the dominant factor affecting the fracture energy of PHRAMs.

### 3.3. Evaluation of Low-Temperature Cracking Resistance

According to the experimental scheme, the fracture energy (*Gf*), fracture toughness (*K*_IC_), and stiffness of the PHRAMs were determined, and the calculated results are presented in Table 9. During the low-temperature cracking resistance test, the specimens exhibited predominantly brittle fracture behavior, making the crack propagation process difficult to capture.

As shown in Table 9, fracture energy generally increased with increasing asphalt binder content, indicating improved low-temperature cracking resistance, and similar trends were observed for the other gradation types. Mixtures with higher asphalt binder contents and finer gradations generally exhibited greater fracture toughness, indicating a greater capacity for energy absorption during fracture and stronger resistance to crack propagation. In contrast, mixtures with lower asphalt binder contents generally exhibited higher stiffness, indicating greater resistance to deformation. However, the variation in stiffness did not exhibit a consistent trend among PHRAMs with different gradations and asphalt binder contents.

To identify a representative indicator for evaluating low-temperature cracking resistance, Pearson correlation analysis was conducted to examine the relationships between low-temperature *Gf* and *K*_IC_ and stiffness. The corresponding correlation coefficients were 0.6039 and −0.1764, respectively. The results indicate that low-temperature *Gf* exhibited a moderate correlation with *K*_IC_, whereas its correlation with stiffness was relatively weak. Considering the physical significance of these indicators and the characteristics of the low-temperature cracking process, low-temperature *Gf* was ultimately selected as the representative indicator for evaluating the low-temperature cracking resistance of PHRAMs.

Based on the calculated low-temperature *Gf* of the PHRAMs, range analysis and ANOVA were performed, and the corresponding results are presented in Table 10 and Table 11, respectively.

As shown in Table 10, as the gradation changed from coarse to fine, the low-temperature *Gf* of the PHRAMs initially increased, subsequently decreased, and then increased again. The low-temperature *Gf* increased with increasing asphalt binder content and mixing time, whereas it exhibited a decreasing trend with increasing RAP preheating temperature. This may be attributed to the relatively long RAP preheating duration of approximately 2 h in the laboratory [30]. Prolonged preheating at elevated temperatures may have intensified the secondary aging of the aged asphalt binder, thereby impairing the low-temperature *Gf* of the mixtures. The range analysis results indicate that the effects of the investigated factors on the low-temperature cracking resistance of PHRAMs followed the order: asphalt content > gradation type > mixing time > RAP preheating temperature, with asphalt binder content exerting the most pronounced influence. Overall, material composition factors had a greater influence than mixing process factors. Among the mixing process factors, mixing time had a greater effect than RAP preheating temperature. This can be mainly attributed to the pronounced increase in the mean fracture energy from 567.850 J/m^2^ to 807.450 J/m^2^ as the asphalt binder content increased from 3.7% to 4.6%. With the objective of achieving a higher low-temperature *Gf*, the optimal combination was determined to be Gradation IV, an asphalt binder content of 4.6%, a mixing time of 180 s, and an RAP preheating temperature of 110 °C.

The ANOVA results presented in Table 11 further indicate that asphalt binder content had a highly significant effect on low-temperature *Gf* (*p* < 0.01), accounting for 81.80% of the total sum of squares and thus representing the dominant factor governing the low-temperature cracking resistance of PHRAMs. In contrast, the effects of gradation type, mixing time, and RAP preheating temperature did not reach statistical significance (*p* > 0.1). Combining the results of the range analysis and ANOVA, asphalt binder content was identified as the most influential factor affecting low-temperature *Gf* within the investigated range of experimental parameters, whereas the effects of the other factors were relatively limited.

### 3.4. Relationships Between RAP Asphalt Mortar Migration, Mixture Characteristics, and Cracking Performance

**Figure 4:**
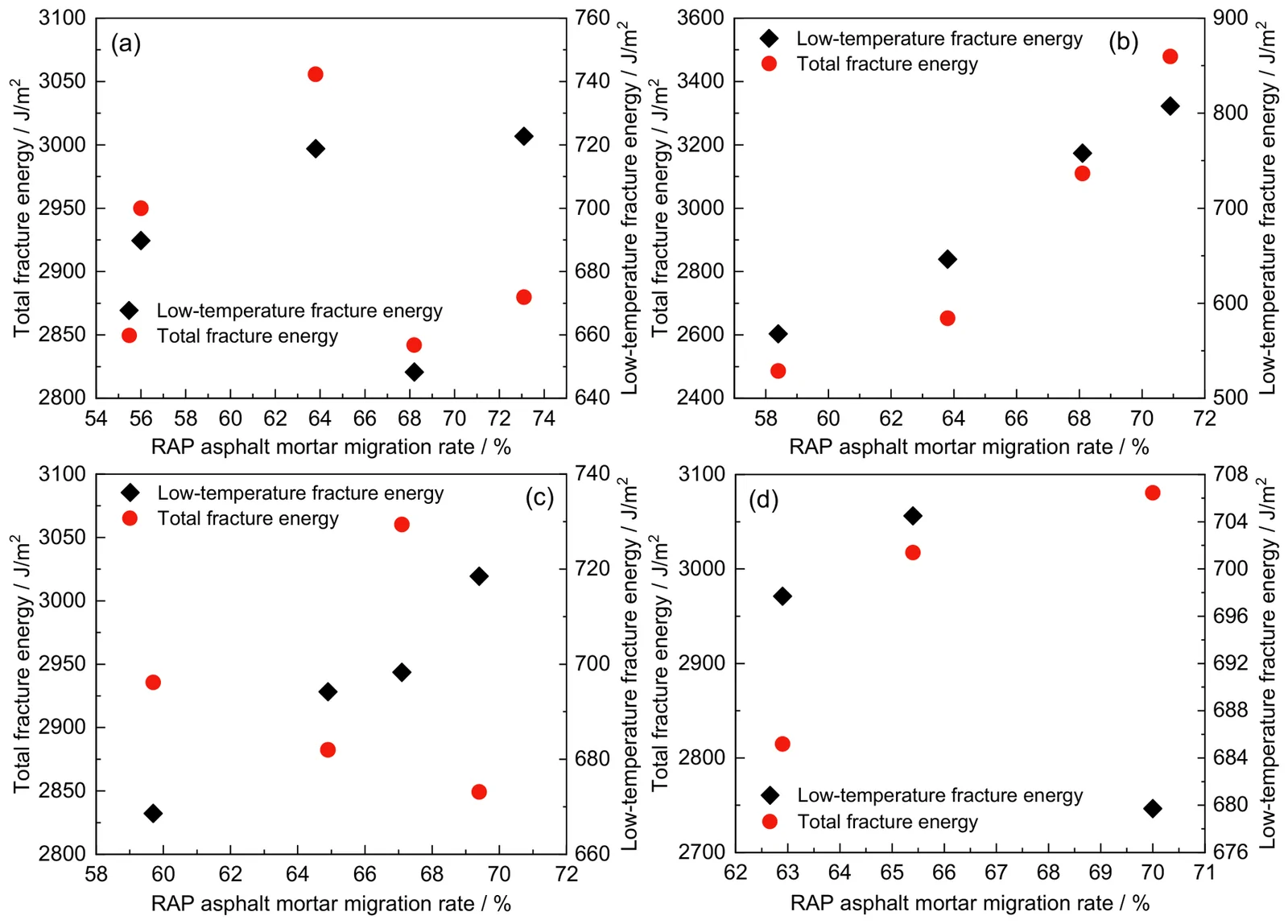
Variation trends between RAP asphalt mortar migration rate and cracking performance under different factors: (**a**) gradation type; (**b**) asphalt content; (**c**) mixing time; (**d**) RAP preheating temperature.

Based on the range analysis results presented in Table 4 and Table 7, and 10, the variation trends between the RAP asphalt mortar migration rate and the crack propagation performance and low-temperature cracking resistance under different influencing factors were plotted, as shown in Figure 4.

The results shown in Figure 4 indicate that the RAP asphalt mortar migration rate is correlated to some extent with the cracking performance of PHRAMs. In particular, variations in asphalt binder content and mixing time resulted in relatively consistent relationships between the RAP asphalt mortar migration rate and both crack propagation resistance and low-temperature cracking resistance. In contrast, no clear monotonic relationships were observed for gradation type and RAP preheating temperature. These findings suggest that RAP asphalt mortar migration is one of the factors affecting the cracking performance of PHRAMs, but it is not the sole dominant factor governing the variation in cracking performance.

**Figure 5:**
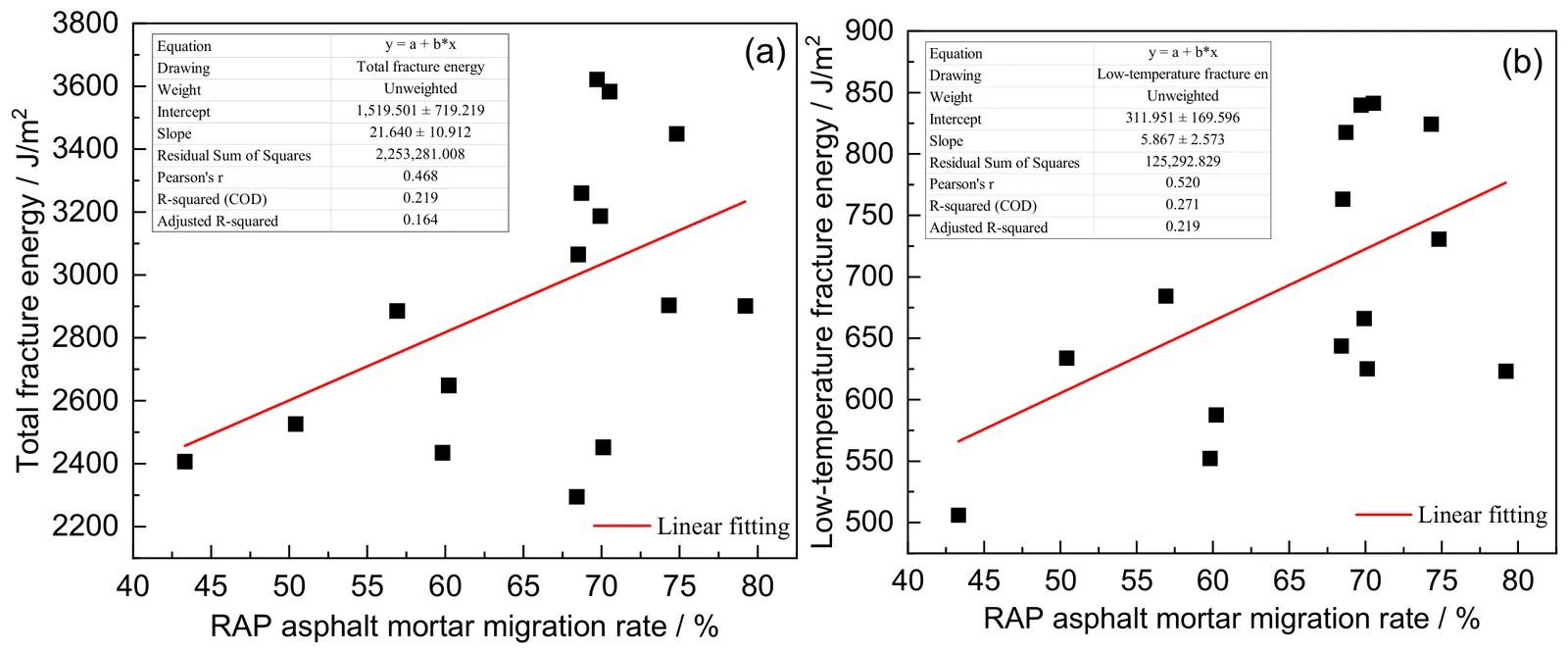
Variation trends of RAP asphalt mortar migration rate and cracking performance of PHRAMs: (**a**) crack propagation performance; (**b**) low-temperature cracking resistance.

Based on the orthogonal experimental results for the RAP asphalt mortar migration rate, crack propagation performance, and low-temperature cracking resistance, the relationships between the RAP asphalt mortar migration rate and total fracture energy and low-temperature fracture energy were plotted, as shown in Figure 5. The results indicate that the RAP asphalt mortar migration rate generally exhibited positive correlations with both crack propagation resistance and low-temperature cracking resistance, suggesting that higher migration indices were associated with higher fracture energies in the pooled observations, although this association does not establish causation. However, the linear correlations were relatively weak, with coefficients of determination (*R*^2^) of 0.219 and 0.271 for crack propagation performance and low-temperature cracking resistance, respectively. The results indicate that the migration index alone provides limited linear explanation of the variation in fracture response.

The relationships shown in Figure 5 should be interpreted as associations rather than evidence of a direct causal effect of RAP mortar migration on cracking performance. Asphalt binder content influences both the migration index and fracture response, and also changes asphalt film thickness and the proportion of aged binder in the total binder system. Gradation and mixing conditions introduce further concurrent changes. The observed association may therefore partly reflect responses to shared experimental parameters. The relatively weak linear relationships indicate limited stand-alone explanatory value of the migration index, but do not establish a negligible causal contribution.

Variations in asphalt binder content and gradation type can alter the asphalt film thickness in PHRAMs, thereby affecting the bonding condition between the asphalt binder and aggregates and, consequently, the cracking resistance of the mixtures. In addition, changes in asphalt binder content modify the proportion of aged asphalt binder in the total binder system, which can further influence the adhesive properties and cracking resistance of the blended asphalt binder. Therefore, based on the orthogonal experimental results obtained in this study, the proportion of aged asphalt binder and asphalt film thickness were calculated for each experimental scheme. In addition, the gradation index was employed to quantitatively characterize the different aggregate gradations [16]. The calculated results are presented in Table 12. Multiple linear regression models were then established using the RAP asphalt mortar migration rate, proportion of aged asphalt binder, asphalt film thickness, and gradation index as independent variables, and total fracture energy and low-temperature fracture energy as dependent variables. The models were used to quantify the effects of these indicators on the cracking resistance of PHRAMs and to identify the key controlling indicators governing crack propagation resistance and low-temperature cracking resistance.

The goodness of fit (*R*^2^), Durbin–Watson test, and normal probability-probability (P–P) plot were employed to evaluate the fitting performance, independence of observations, and normality of residuals of the multiple linear regression models, respectively. In the models, *x*_1_, *x*_2_, *x*_3_, and *x*_4_ represent the RAP asphalt mortar migration rate, proportion of aged asphalt binder, asphalt film thickness, and gradation index, respectively, while yc and ylc represent the total fracture energy and low-temperature fracture energy, respectively. The corresponding results of the multiple linear regression analysis are presented in Table 13.

As shown in Table 13, the R2 for the regression models of total fracture energy for crack propagation and low-temperature fracture energy were 0.892 and 0.828, respectively, indicating good model fitting performance. The Durbin–Watson statistics of the two models were 2.943 and 1.836, respectively, suggesting that the observations generally exhibited satisfactory independence [16]. In addition, the data points in the P–P plots were distributed approximately along the 45° reference line, indicating that the model residuals approximately followed a normal distribution. Overall, the established multiple linear regression models exhibited good fitting performance and statistical applicability.

The parameters obtained from the multiple linear regression models were used to evaluate the effects of the individual indicators on the total fracture energy for crack propagation and low-temperature fracture energy, and the corresponding results are presented in Table 14. Among these parameters, the standardized regression coefficient Beta eliminates the influence of differences in variable dimensions and scales, thereby enabling direct comparison of the relative effects of the different indicators. Therefore, the absolute value of Beta was used as the basis for evaluating the relative influence weight of each indicator.

As shown in Table 14, the absolute values of the *β*-values in both the total fracture energy and low-temperature fracture energy regression models followed the same order: asphalt film thickness > gradation index > proportion of aged asphalt binder > RAP asphalt mortar migration rate. This ranking suggests that asphalt film thickness and gradation index were more strongly associated with the cracking performance of the PHRAMs within the fitted models. The positive *β*-value for asphalt film thickness and the negative *β*-value for gradation index indicate positive and negative relationships with cracking performance, respectively. In contrast, the relatively small *β*-value of the RAP asphalt mortar migration rate suggests that its independent explanatory effect was limited.

It should be noted that none of the variables in either model reached the 0.05 significance level. Moreover, except for the RAP asphalt mortar migration rate, the VIF values of the remaining variables were substantially greater than 10, indicating strong multicollinearity among the independent variables. Therefore, the standardized regression coefficients should not be interpreted directly as measures of the independent contribution of each indicator. Considering the regression results together with the preceding experimental analyses, asphalt film thickness and gradation index can be regarded as potential key indicators closely associated with both crack propagation resistance and low-temperature cracking resistance of PHRAMs. Meanwhile, although RAP asphalt mortar migration was related to cracking performance to some extent, its independent effect was relatively limited when multiple factors were considered simultaneously. These findings indicate that the cracking performance of PHRAMs is governed by the combined effects and interactions of multiple factors.

The findings of this study should be interpreted in light of the limitations of its experimental design. Specifically, the RAP content was fixed at 40% by mass of the total aggregate, although variations in RAP content may alter the amount of aged mortar, the proportions of reclaimed and virgin materials, and their interactions during mixing. These changes may further influence the effects of gradation, binder content, mixing time, and RAP preheating temperature on mixture performance. Since RAP content was not varied, its individual and interaction effects, as well as its optimal content, could not be determined. Therefore, the observed factor effects and relationships between the migration proxy and fracture performance are limited to the investigated RAP content, material system, and experimental conditions, requiring further validation before being generalized to other mixtures.

In addition to these limitations in generalizability, the statistical relationships should also be interpreted with caution. The weak bivariate correlations between the migration proxy and fracture performance suggest that the migration proxy alone may not fully explain the observed fracture response, but do not establish the relative importance of other mixture characteristics. Therefore, the regression analysis should be regarded as exploratory rather than a basis for ranking independent contributions. Although asphalt film thickness, aged binder proportion, and gradation may also influence fracture performance, their individual contributions and underlying physical mechanisms require further experimental verification.

### 3.5. Regulation Strategy of RAP Particle Dispersion Behavior Under Cracking Performance Constraints

Material composition and mixing process parameters not only affect the cracking performance of PHRAMs but also alter the dispersion characteristics of RAP particles during hot mixing. The results indicate that the RAP asphalt mortar migration rate can be used to characterize the dispersion behavior of RAP particles and, together with asphalt film thickness, proportion of aged asphalt binder, and gradation index, influences the cracking performance of the mixture. By establishing multiple linear or nonlinear models relating these indicators to cracking performance, a reasonable control level for the RAP asphalt mortar migration rate can be determined under a specified cracking performance target. On this basis, the corresponding ranges of material composition and mixing process parameters can be inversely determined.

**Figure 6:**
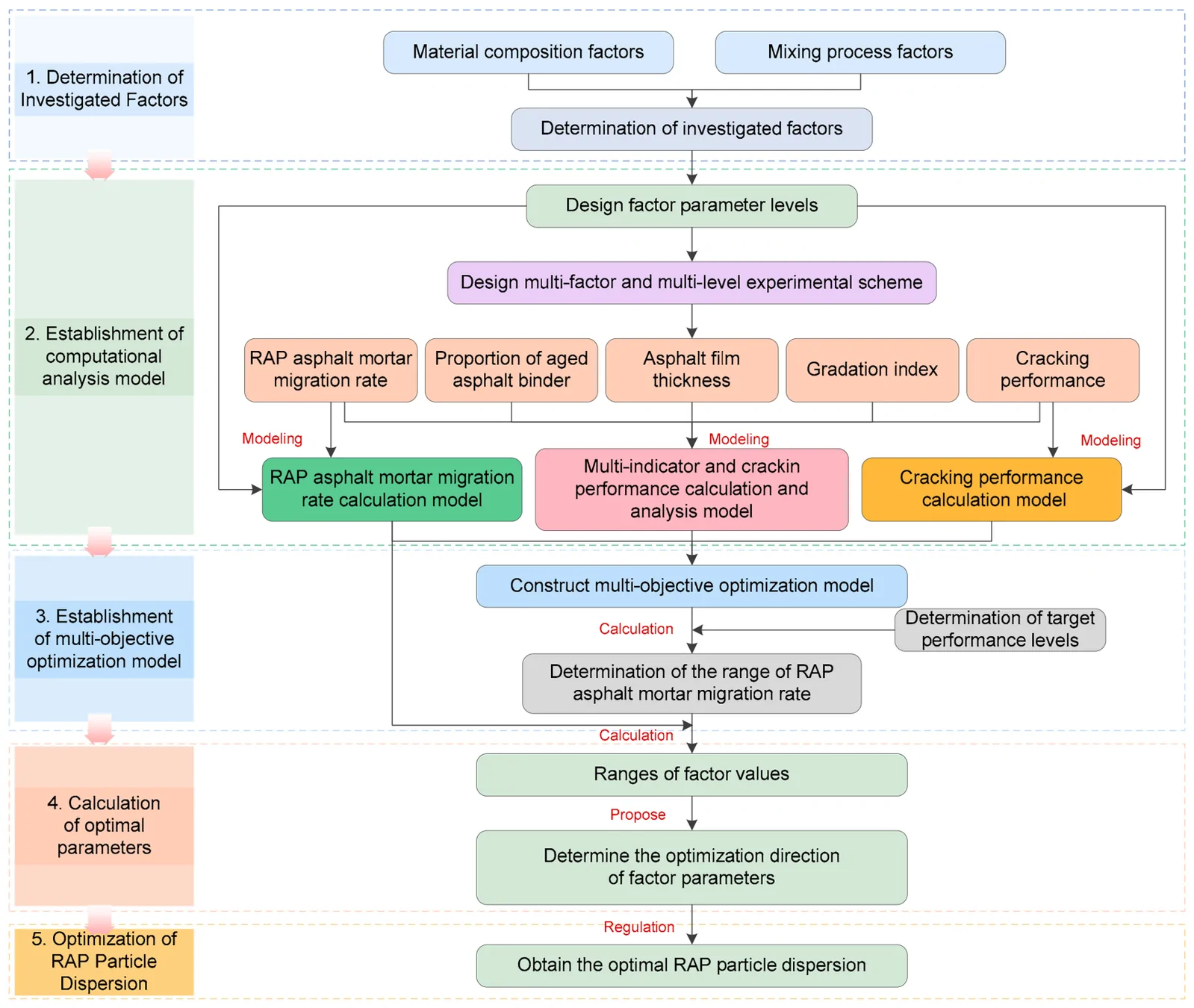
Regulation strategy for RAP particle dispersion behavior.

Accordingly, a method for regulating RAP particle dispersion behavior was developed with cracking performance as the constraint. First, a relationship model between the RAP asphalt mortar migration rate and the material composition and mixing process parameters is established. This model can be developed using orthogonal experimental design, response surface methodology, or intelligent algorithms based on a sufficiently large experimental dataset. Second, relationship models are established between indicators such as asphalt film thickness and proportion of aged asphalt binder and the material composition and mixing process parameters. Subsequently, a multivariate analysis model is constructed to relate the RAP asphalt mortar migration rate, asphalt film thickness, proportion of aged asphalt binder, and gradation index to cracking performance. Finally, multi-objective optimization is performed to determine the appropriate control level of RAP asphalt mortar migration and the reasonable ranges of material composition and mixing process parameters while satisfying the specified cracking performance requirements. The corresponding technical framework is illustrated in Figure 6.

Accordingly, the proposed optimization framework should be considered a conceptual approach for future process optimization rather than an established regulation methodology. Although this study provides experimental insights into the relationships between mixing conditions and mixture performance, the development of a comprehensive optimization framework requires a larger dataset, reliable predictive models, appropriate optimization algorithms, and independent experimental validation. Future studies should further expand the experimental scope and validate the optimized parameters to improve the generalizability and practical applicability of the proposed framework.

## 4. Conclusions

(1) The RAP asphalt mortar migration rate was proposed as an indicator to characterize the dispersion behavior of RAP particles during hot mixing, and the effects of gradation type, asphalt binder content, mixing time, and RAP preheating temperature were investigated. The results showed that the influence of these factors on the RAP asphalt mortar migration rate followed the order: gradation type > asphalt binder content > mixing time > RAP preheating temperature. Among them, gradation type and asphalt binder content had highly significant effects, whereas the effects of mixing time and RAP preheating temperature were relatively limited.

(2) The factors affecting the crack propagation resistance of PHRAMs were ranked as follows, asphalt binder content > RAP preheating temperature > gradation type > mixing time, among which asphalt binder content and RAP preheating temperature had highly significant effects. For low-temperature cracking resistance, the influence of the investigated factors followed the order asphalt binder content > gradation type > mixing time > RAP preheating temperature, with only asphalt binder content reaching a highly significant level. Overall, considering the cracking performance under different temperature conditions, asphalt binder content consistently emerged as the key influencing factor.

(3) RAP asphalt mortar migration alone did not adequately explain the variation in cracking performance within the investigated experimental domain. Changes in material composition and mixing conditions also altered asphalt film thickness, gradation characteristics, and the proportion of aged binder. These descriptors should therefore be considered together when interpreting fracture response. Strong multicollinearity in the regression models limits the attribution of independent effects to individual descriptors. However, from a general perspective, the influence of asphalt film thickness and gradation type is stronger than that of the RAP asphalt mortar migration index.

(4) A conceptual framework was proposed for regulating RAP particle dispersion subject to cracking-performance requirements. The framework links material composition and mixing conditions to mortar migration and mixture characteristics, and subsequently to fracture response. Further experimental data and independent validation are required before it can be used to determine reliable parameter ranges for production.

(5) The generalizability of the findings is limited by both the experimental design and the scope of cracking characterization. As RAP content was fixed at 40%, its effects and interactions with material composition and mixing conditions were not evaluated, and an optimum RAP content could not be identified. In addition, cracking behavior was assessed only through SCB tests at the investigated intermediate and low temperatures under specified loading conditions. Since these tests share the same specimen geometry and bending configuration, the results reflect fracture responses under the tested conditions rather than comprehensive cracking performance. Accordingly, the observed relationships between RAP mortar migration and fracture performance, as well as potential parameter recommendations, require further validation before being extended to other RAP contents or cracking conditions. Future studies should investigate a broader range of RAP contents and their interactions, incorporate complementary tests addressing fatigue, thermally induced cracking, and reflective cracking, and validate the findings against field performance.

## Figures and Tables

**Table 1:** Test results for the basic properties of RAP.

Material Type	Test Parameter	Test Result	Test Method
RAP	Moisture Content (%)	0.42	T 0307 and T 0334
	Sand Equivalent (%)	78.9	—
Asphalt Binder Recovered from RAP	Penetration at 25 °C (0.1 mm)	31.2	T 0604
	Softening Point (°C)	68.9	T 0606
	Ductility at 5 °C (cm)	Brittle fracture	T 0605
	Viscosity at 135 °C (Pa·s)	2.035	T 0620
Coarse Aggregate in RAP	Crushing Value (%)	13.2	T 0316
	Flat and Elongated Particle Content (%)	14.3	T 0312
Fine Aggregate in RAP	Angularity	30.8	T 0345

**Table 2:** Test results for the basic properties of aggregates.

Aggregate Size	Test Item		Test Result	Test Method
10–20 mm	Apparent Relative Density		2.726	T 0304
	Water Absorption (%)		0.8	T 0304
	Flat and Elongated Particle Content (%)		10.2	T 0312
	Crushing Value (%)		13.4	T 0316
	Los Angeles Abrasion Loss (%)		14.6	T 0317
	Adhesion		5 Level	T 0616
5–10 mm	Apparent Relative Density		2.711	T 0304
	Water Absorption (%)		1.1	T 0304
	Flat and Elongated Particle Content (%)		16.3	T 0312
	Los Angeles Abrasion Loss (%)		15.2	T 0317
	Adhesion		5 Level	T 0616
0–5 mm	Apparent Relative Density		2.721	T 0328
	Sand Equivalent (%)		75	T 0334
Mineral filler	Apparent Density (g/cm^3^)		2.702	T 0352
	Apparent Relative Density		2.709	
	Particle Size Distribution (%)	<0.6 mm	100	T 0351
		<0.15 mm	99.8	
		<0.075 mm	96.7	

**Table 3:** Orthogonal experimental design [30].

Experimental Scheme	Gradation Type	Asphalt Content (%)	Mixing Time (s)	RAP Preheating Temperature (°C)
1	Gradation I	3.7	60	90
2	Gradation I	4.3	150	110
3	Gradation I	4	120	90
4	Gradation I	4.6	180	130
5	Gradation II	4.6	150	90
6	Gradation II	4	60	110
7	Gradation II	4.3	180	90
8	Gradation II	3.7	120	130
9	Gradation III	4	180	90
10	Gradation III	4.6	120	110
11	Gradation III	3.7	150	90
12	Gradation III	4.3	60	130
13	Gradation IV	4.3	120	90
14	Gradation IV	3.7	180	110
15	Gradation IV	4.6	60	90
16	Gradation IV	4	150	130

**Table 4:** Range analysis results for RAP asphalt mortar migration rate.

Item	Gradation Type	Asphalt Content (%)	Mixing Time (s)	RAP Preheating Temperature (°C)
$\bar{K_{1}}$	56.0	58.4	59.7	62.9
$\bar{K_{2}}$	63.8	63.8	64.9	65.4
$\bar{K_{3}}$	68.2	68.1	67.1	70.0
$\bar{K_{4}}$	73.1	70.9	69.4	—
Range	17.1	12.6	9.7	7.1
Factor Ranking	Gradation Type > Asphalt Content > Mixing Time > RAP Preheating Temperature			
Optimal Combination	Gradation IV	4.60%	180 s	130 °C

Note: $\bar{K_{j}}$ represents the mean value of the experimental results at level (*j*). A larger range indicates a greater influence of the corresponding factor, whereas a smaller range indicates a weaker influence.

**Table 5:** ANOVA results for RAP asphalt mortar migration rate.

Source of Variation	Sum of Squares (*SS*)	df	Mean Square (*MS*)	*F*-Value	*p*-Value	Significance
Gradation Type	634.402	3	211.467	44.914	0.002	***
Asphalt Content	360.482	3	120.161	25.521	0.005	***
Mixing Time	205.012	3	68.337	14.514	0.013	**
RAP Preheating Temperature	133.046	2	66.523	14.129	0.015	**
Error	18.833	4	4.708	—	—	—
Corrected Total	1351.774	15	—	—	—	—

Note: * indicates a certain effect at (*p* < 0.1), ** indicates a significant effect at (*p* < 0.05), and *** indicates a highly significant effect at (*p* < 0.01).

**Table 6:** Test results for crack propagation performance of PHRAMs.

Experimental Scheme	*F*_max_ (kN)	$G_{f}^{I}$ (J/m^2^)	$G_{f}^{\mathrm{II}}$ (J/m^2^)	*Gf* (J/m^2^)	*m* (kN/mm)	*FI*
1	4.93	763	1644.7	2407.7	−3.376	7.2
2	4.11	972	2309.9	3281.9	−2.049	16.1
3	3.85	903	1623.9	2526.9	−2.132	12
4	3.52	1329.9	2253.7	3583.6	−1.913	18.7
5	4.63	1248.8	2373	3621.8	−2.593	14.2
6	4.49	1178.6	1707.6	2886.1	−4.874	5.9
7	4.96	1062.8	2002.6	3065.5	−3.667	8.4
8	5.1	1138.9	1510.6	2649.4	−4.687	5.7
9	4.79	966.5	1328.8	2295.3	−5.073	4.6
10	4.73	1018	2431.4	3449.4	−2.601	13.8
11	4.75	902.6	1532.9	2435.5	−4.802	5.1
12	4.67	1122.4	2065.5	3187.9	−3.095	10.4
13	4.92	1213.8	1690.3	2904.1	−4.743	6.2
14	4.59	900.6	1551.9	2452.5	−4.610	5.3
15	4.97	1170.3	2090.2	3260.6	−3.853	8.5
16	5.61	1055	1847.1	2902.1	−6.049	4.8

**Table 7:** Range analysis results for total fracture energy (*Gf*).

Item	Gradation Type	Asphalt Content (%)	Mixing Time (s)	RAP Preheating Temperature (°C)
$\bar{K_{1}}$	2950.0	2486.3	2935.6	2814.7
$\bar{K_{2}}$	3055.7	2652.6	2882.4	3017.5
$\bar{K_{3}}$	2842.0	3109.8	3060.3	3080.7
$\bar{K_{4}}$	2879.8	3478.8	2849.2	—
Range	213.7	992.5	211.1	266
Factor Ranking	Asphalt Content > RAP Preheating Temperature > Gradation Type > Mixing Time			
Optimal Combination	Gradation II	4.60%	150 s	130 °C

**Table 8:** ANOVA results for total fracture energy (*Gf*).

Source of Variation	Sum of Squares (*SS*)	df	Mean Square (*MS*)	*F*-Value	*p*-Value	Significance
Gradation Type	105,777.11	3	35,259.04	7.1	0.0443	**
Asphalt Content	2,429,642.54	3	809,880.85	163.09	0.000123	***
Mixing Time	103,147.78	3	34,382.59	6.924	0.0462	**
RAP Preheating Temperature	227,851.22	2	113,925.61	22.942	0.00643	***
Error	19,863.45	4	4965.86	—	—	—
Corrected Total	2,886,282.09	15	—	—	—	—

Note: * indicates a certain effect at (*p* < 0.1), ** indicates a significant effect at (*p* < 0.05), and *** indicates a highly significant effect at (*p* < 0.01).

**Table 9:** Test results for low-temperature cracking resistance of PHRAMs.

Experimental Scheme	*Gf* (J/m^2^)	*K*_IC_ (MPa·m^0.5^)	Stiffness (kN/mm)
1	506.2	1.054	12.9
2	777.6	1.285	16.9
3	634.0	1.198	19.1
4	841.4	1.325	19.7
5	839.8	1.375	16.1
6	684.4	1.312	21.5
7	763.4	1.185	16.7
8	587.7	1.124	21.7
9	643.8	1.307	21.4
10	730.9	1.185	18.4
11	552.3	1.264	22.7
12	666.3	1.328	20.7
13	824.5	1.406	18.6
14	625.2	1.358	24.1
15	817.7	1.357	21
16	623.3	1.284	20.4

**Table 10:** Range analysis results for low-temperature fracture energy (*Gf*).

Item	Gradation Type	Asphalt Content (%)	Mixing Time (s)	RAP Preheating Temperature (°C)
$\bar{K_{1}}$	689.8	567.8	668.6	697.7
$\bar{K_{2}}$	718.8	646.4	694.2	704.5
$\bar{K_{3}}$	648.3	757.9	698.3	679.7
$\bar{K_{4}}$	722.7	807.5	718.5	—
Range	74.4	239.6	49.8	24.8
Factor Ranking	Asphalt Content > Gradation Type > Mixing Time > RAP Preheating Temperature			
Optimal Combination	Gradation IV	4.60%	180 s	110 °C

**Table 11:** ANOVA results for low-temperature fracture energy (*Gf*).

Source of Variation	Sum of Squares (*SS*)	df	Mean Square (*MS*)	*F*-Value	*p*-Value	Significance
Gradation Type	14,156.39	3	4718.80	1.758	0.2936	—
Asphalt Content	140,556.73	3	46,852.24	17.46	0.0092	***
Mixing Time	5021.11	3	1673.70	0.624	0.6362	—
RAP Preheating Temperature	1361.05	2	680.523	0.254	0.7876	—
Error	10,733.69	4	2683.42	—	—	—
Corrected Total	171,828.97	15	—	—	—	—

Note: * indicates a certain effect at (*p* < 0.1), ** indicates a significant effect at (*p* < 0.05), and *** indicates a highly significant effect at (*p* < 0.01).

**Table 12:** Calculated parameters of PHRAMs in the orthogonal experiment.

Experimental Scheme	RAP Asphalt Mortar Migration Rate (%)	Proportion of Aged Asphalt Binder (%)	Asphalt Film Thickness (μm)	Gradation Index (%)
1	43.3	44.25	9.02	76.8
2	59.6	37.84	10.48	76.8
3	50.4	40.81	9.75	76.8
4	70.5	35.26	11.21	76.8
5	69.7	35.26	10.18	65.3
6	56.9	40.81	8.85	65.3
7	68.5	37.84	9.51	65.3
8	60.2	44.25	8.18	65.3
9	68.4	40.81	7.98	57.2
10	74.8	35.26	9.17	57.2
11	59.8	44.25	7.38	57.2
12	69.9	37.84	8.57	57.2
13	74.3	37.84	7.80	50.2
14	70.1	44.25	6.71	50.2
15	68.7	35.26	8.35	50.2
16	79.2	40.81	7.26	50.2

**Table 13:** Results of multiple linear regression model analysis.

Indicator		Result
Total fracture energy	Regression Model	*y_c_* = −2173.007 + 3.709*x*_1_ + 58.739*x*_2_ + 790.122*x*_3_ − 70.429*x*_4_
	*R* ^2^	0.869
	Durbin–Watson Test	2.541
	Normal P–P Plot	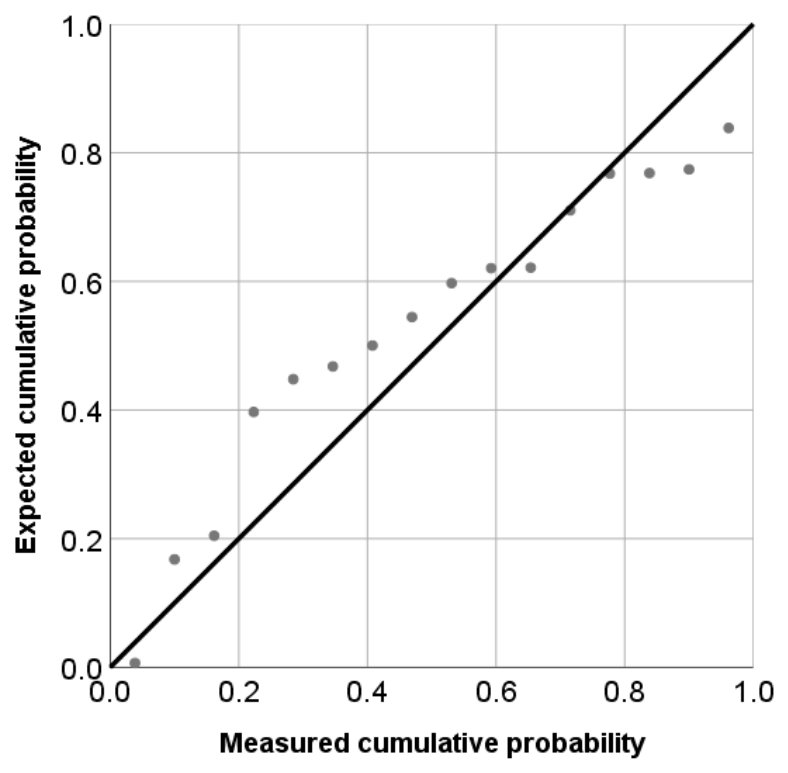
Low-temperature fracture energy	Regression Model	*y_lc_* = 435.256 + 0.234*x*_1_ − 1.862*x*_2_ + 121.101*x*_3_ − 11.939*x*_4_
	*R* ^2^	0.828
	Durbin–Watson Test	1.836
	Normal P–P Plot	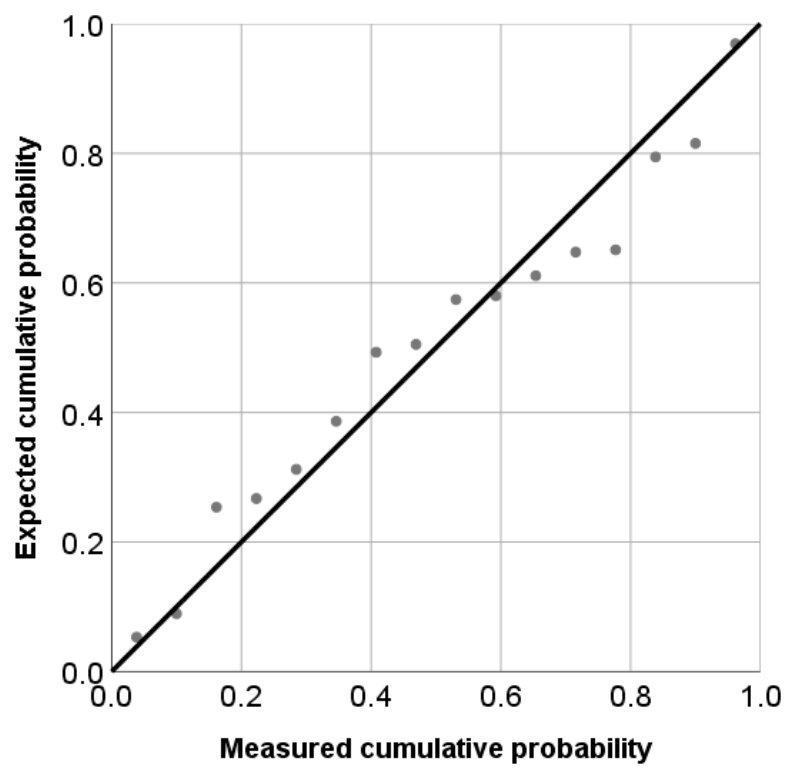

**Table 14:** Multiple linear regression parameter analysis results.

Indicator	Parameter	Unstandardized Coefficient		Standardized Coefficient		*p*-Value	Collinearity Statistics	
		Value	Standard Error	*β*-Value	*t*-Value		Tolerance	VIF
Total fracture energy	Constant	−2173.01	4480.145	—	−0.485	0.637	—	—
	*x*_1_	3.709	11.316	0.08	0.328	0.749	0.199	5.02
	*x*_2_	58.739	90.983	0.464	0.646	0.532	0.023	43.219
	*x*_3_	790.122	458.999	2.24	1.721	0.113	0.007	141.679
	*x*_4_	−70.429	49.072	−1.641	−1.435	0.179	0.009	109.409
Low-temperature fracture energy	Constant	435.256	1251.785	—	0.348	0.735	—	—
	*x*_1_	0.234	3.162	0.021	0.074	0.942	0.199	5.02
	*x*_2_	−1.862	25.421	−0.06	−0.073	0.943	0.023	43.219
	*x*_3_	121.101	128.248	1.407	0.944	0.365	0.007	141.679
	*x*_4_	−11.939	13.711	−1.14	−0.871	0.403	0.009	109.409

Note: *x*_1_, *x*_2_, *x*_3_, and *x*_4_ denote the RAP asphalt mortar migration rate, proportion of aged asphalt binder, asphalt film thickness, and gradation index, respectively; VIF denotes the variance inflation factor.

## Data Availability

The original contributions presented in this study are included in the article. Further inquiries can be directed to the corresponding author.
